# Risk factors for diabetic foot complications among patients with type 2 diabetes in Austria–A registry‐based retrospective cohort study

**DOI:** 10.1002/edm2.286

**Published:** 2021-07-13

**Authors:** Sophia Rossboth, Benedikt Rossboth, Hans Schoenherr, Monika Lechleitner, Willi Oberaigner

**Affiliations:** ^1^ Department of Public Health Research Unit for Diabetes Epidemiology Health Services Research and Health Technology Assessment Medical Informatics and Technology UMIT‐Private University for Health Sciences Hall i.T. Austria; ^2^ Department of Internal Medicine St. Vinzenz Hospital Zams Zams Austria; ^3^ Department of Internal Medicine Hospital Hochzirl Zirl Austria

**Keywords:** diabetic foot, real‐world data, risk factors, type 2 diabetes mellitus

## Abstract

**Aims:**

Diabetic foot complications, a serious consequence of diabetes mellitus, are associated with a tremendous burden on both individual patients and health care systems. Since prevention strategies may reduce the incidence of this complication, identification of risk factors in large longitudinal studies is essential to optimize early detection and personalized screening of patients at increased risk.

**Materials and methods:**

We conducted a registry‐based retrospective cohort study using data from 10,688 patients with type 2 diabetes mellitus aged ≥18 years. Cox regression models were used to identify risk factors for foot complications while adjusting for potential confounders.

**Results:**

We observed 140 diabetic foot complications in our patient cohort. The multivariate Cox regression model revealed neuropathy, peripheral arterial disease and male gender as being positively associated with foot complications. The same effect was detected for nephropathy in the time >10 years after T2DM diagnosis. For higher age at diagnosis and use of insulin, however, a negative association was retrieved.

**Conclusion:**

Male gender and several diabetes‐related comorbidities were identified as risk factors for subsequent initial foot complications in patients with type 2 diabetes mellitus. These findings suggest that personalized early detection of patients at increased risk might be feasible by using information on demographics, medical history and comorbidities.

## Graphical abstract

A registry‐based cohort study was conducted on 10,688 patients with type 2 diabetes mellitus. The time‐dependent Cox regression analysis identified neuropathy, peripheral arterial disease, nephropathy and male gender to be associated with an increased risk of subsequent foot complications, while higher age at diagnosis and use of insulin was associated with decreased risk. Personalized screening for patients at increased risk is suggested to be possible by using readily available medical information.

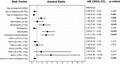

## 1 INTRODUCTION

Diabetes mellitus (DM) represents one of the major public health concerns worldwide. Global estimates indicate that 463 million people aged 18–99 years are currently affected by DM and this number is projected to increase to over 700 million people by 2045. The rising prevalence of diabetes can be explained by ageing, population growth and lifestyle alterations.^1^, ^2^, ^3^ Diabetic patients face a high risk of subsequent adverse health conditions associated with severe morbidity, shortened life expectancy and increased health care expenditures.^2^, ^4^ In accordance with the rising number of patients with DM, the incidences of those late complications are also increasing. The diabetic foot (DF) syndrome, which constitutes one of the most severe late complications, affects up to 25% of adults with diabetes during their lifetime.^5^ Of those patients, 20% will require lower extremity amputation,^6^which amounts to a lower limb being lost due to a DF every 20 s globally.^7^ According to the International Working Group on the Diabetic Foot (IWGDF), the DF syndrome which encompasses several diagnoses such as foot ulcers, Charcot foot and lower extremity amputations, is defined as ‘Infection, ulceration, or destruction of tissues of the foot of a person with currently or previously diagnosed diabetes mellitus, usually accompanied by neuropathy and/or peripheral arterial disease in the lower extremity.’^8^ DF complications are associated with increased mortality, which was shown by different large studies across the globe.^9^, ^10^, ^11^ The complications are furthermore associated with lower quality of life^12^ and tremendous medical care costs^2^, ^4^ that increase with time.^13^ The health expenditures for patients with diabetic foot ulcers are five times higher compared to diabetic patients without foot complications,^2^ rendering diabetic foot ulcers and amputations the most expensive diabetic late complication in terms of hospital costs.^14^

However, foot ulcerations are suggested to be highly preventable.^15^, ^16^ From a public health perspective, a crucial pillar in the prevention of DF is to identify the role of risk factors to facilitate the early detection of patients at high risk for subsequent foot complications.^17^ In a recent and comprehensive systematic review, the results on risk factors for DF in patients with T2DM were brought together: A relatively consistent positive association was retrieved between male gender, duration of diabetes, poor glycaemic control, smoking, height, neuropathy, retinopathy, nephropathyand insulin use with subsequent DF development. However, inconsistent results were obtained for example, for age, hypertension, dyslipidaemia and body mass index. Although predefined stringent inclusion and exclusion criteria were applied in this systematic review, a large heterogeneity remained among the included studies regarding study design and patient populations.^18^ This heterogeneity and the inconsistencies in the results of the included studies highlights the need for further research on risk factor profiles for DF in different regions of the world to aid the improvement of prevention and early detection strategies and increase the patients’ quality of life, while reducing the financial burden for the public health system.^19^ The significant value of state‐of‐the art prevention and treatment of DF to reduce the substantial amputation rate was emphasized by the IWGDF.^20^

Chronic diseases, such as diabetes and associated late complications, can be studied reliably by the analysis of disease registry data.^19^ Since data in population‐based registries are collected in a comprehensive manner without stringent in‐ and exclusion criteria, results of its analyses are highly generalizable and applicable to a wide range of patients.^21^

The purpose of this study was to determine which factors are associated with an increased risk for subsequent diabetic foot complications in patients with type 2 diabetes mellitus (T2DM) using real‐world data from a large cohort of patients. To this end, data from the Diabetes Registry of Tyrol (DRT) were used. This is one of the largest diabetes registries in Europe and represents all hospitals and several outpatient practices in one out of nine federal states in Austria.

## 2 SUBJECTS, MATERIALS AND METHODS

### 2.1 Study population

The DRT was established in 2006 aiming to measure and improve the quality of care for diabetic patients in Tyrol. Data are collected in ten participating hospital sites covering all hospitals in Tyrol as well as eight outpatient practices of specialists of internal medicine. All patients with newly diagnosed T1DM, T2DM and gestational diabetes mellitus, but also prevalent diabetes patients who attend an outpatient department, are collected in the DRT. After patients undergo a comprehensive clinical assessment during the first visit at one of the participating sites, they are invited to return for quarterly visits. Until 2019, data on more than 24,000 diabetic patients were collected within this registry.^22^, ^23^ The registration is performed within the hospital information systems, which incorporate demographic data, diabetes‐related clinical and biochemical parameters and data on late complications related to diabetes.^23^ After pseudonymization, the data are transferred to the DRT. This allows linkage of data for a specific patient registered in different departments and guarantees data confidentiality.

### 2.2 Case identification and definitions

The following three tests are used for establishing the diagnosis of diabetes mellitus: (i) ≥126 mg/dl (≥7.0 mmol/L) of plasma glucose in a fasting state, (ii) ≥200 mg/dl (≥11.1 mmol/L) either in a nonfasting state or two hours after oral intake of 75 grams of glucose or (iii) a HbA1c value of ≥6.5%. An unequivocal diagnosis requires either two different tests, or one test performed on two separate days.^24^

In addition to the year of diabetes diagnosis and the patient's demographic data, height and smoking status are documented at entry in the registry. At each subsequent visit, data on weight, physical activity (defined as at least 2.5 h per week) and current diabetes treatment are updated, and the values for blood pressure and HbA1c are assessed.

Retinopathy is diagnosed according to the guidelines provided by the Austrian ophthalmologist association, which are based on the international retinopathy severity scales.^25^ Neuropathy is assessed based on a monofilament test performed by the physician in charge according to local standard of care, and the diagnosis for nephropathy requires positive albumin results (≥30 mg/24 h) at two subsequent visits or a single visit with decreased glomerular filtrate rate of <60 ml/min as calculated using the MDRD 4‐variable equation. In addition, cardiovascular diseases are assessed if a diagnosis for acute coronary syndrome or angiography is given, and cerebrovascular diseases are assessed if a diagnosis for minor and major strokes including transient ischaemic attacks is present. Peripheral arterial disease is diagnosed according to the guidelines of the Austrian Diabetes Association^26^ via clinical examination (pulse status in the legs with ankle‐brachial index <0.9) and if required by sonography and/or angiography. The definition of diabetic foot complications requires at least a clinical diagnosis of a foot ulcer. Amputation is defined as any nontraumatic amputation in the lower extremity due to diabetes. For all late complications, the year of the first occurrence is collected in the registry.

The retrospective cohort study was designed and reported in accordance with the Strengthening the Reporting of Observational Studies in Epidemiology (STROBE) guidelines.^27^ The following inclusion criteria were defined for the patient population included in the analysis: (1) established diagnosis of T2DM; (2) known year of diabetes diagnosis; (3) patient's age at diagnosis of at least 18 years; (4) no present or prior foot ulcer at the first visit of a patient in the registry; (5) patients with complete data sets concerning relevant demographic and clinical data that were considered potential risk factors for DF. Thereby, patients with other types of diabetes (e.g. T1DM or gestational diabetes) and patients younger than 18 years at diagnosis were excluded. Data collected between 2006 and 2019 were included in the analysis.

As preceding risk factors for the initial development of DF were assessed, only data collected at visits before the year of DF diagnosis were included. The variables collected at each visit were aggregated to yield data values on patient level. For HbA1c, systolic and diastolic blood pressure and weight, the mean values were used. The patient's smoking status at time of diagnosis was classified as ‘active smoker’ or ‘ex‐ or never‐smoker’. Physical activity was considered applicable if the patient responded positively to being active for ≥2.5 h per week at least at one visit collected in the registry.

Based on the commonly used classification of age groups within the Diabetes Registry Tyrol, the patient's age at diagnosis was categorized into three groups: ≤50 years, >50 and ≤70 years, and >70 years of age. Hypertension was considered evident if the mean of all systolic or diastolic blood pressure readings prior to DF development or until last visit was at or above 140 or 90 mmHg, respectively. The body mass index (BMI) was calculated, and obesity was defined as a BMI of 30 kg/m² or more in accordance with the definition by the World Health Organization (WHO).^28^ For each patient, the mean of all HbA1c values was calculated and used to assign the respective patient to one of three groups: <6.5%, 6.5%–9.0%, and >9.0%. The boundaries were chosen due to different approaches to antihyperglycaemic treatment as specified in the guidelines of the Austrian Diabetes Association,^29^The use of insulin or insulin analogues was assessed as potential risk factor for DF. Thereby, a distinction was made between patients for whom this form of treatment was documented at least at one visit, and patients who have never been treated in this way. Additionally, it was assessed, if the patients participated in an educational programme, and if the conduction of foot inspections was documented for at least one visit.

The following pre‐existing late complications were considered as potential risk factors for DF and assessed as binary variables: nephropathy, retinopathy, neuropathy, myocardial infarction, stroke, peripheral arterial disease and coronary bypass/percutaneous transluminal coronary angioplasty (PTCA).

Since the need for lower extremity amputation usually follows a preceding DF complication such as a foot ulcer, the year of the first documented amputation was usually after the year of the first DF diagnosis. However, in rare cases in which no DF diagnosis preceded a present year of amputation, the year of amputation was defined as the year of the DF diagnosis.

### 2.3 Statistical analysis

While categorical variables were reported in proportions, continuous variables were described as means ± standard deviations. For the comparison of variables between the cohort of patients with DF and the cohort of patients without DF, Chi square (χ²) and Mann–Whitney *U* tests were used for categorical and continuous variables, respectively. *p*‐Values <.05 were considered statistically significant.

Cox regression analysis was used to analyse the association between potential risk factors and subsequent DF complications. To this end, the time from initial diabetes diagnosis to incidence of DF was assessed, while the end of follow‐up was considered the censoring event. As only 170 patients (1.6% of all patients) exceeded 35 years of follow‐up time, the data were truncated at this point of time. The proportional hazard assumption (PHA) was reviewed using two graphical approaches (visual inspection of log‐log curves and fit of a univariate Cox regression model against the empirical survival curves) and a test based on Schoenfeld residuals. Violations of the PHA were observed for nephropathy, stroke and HbA1c. In order to resolve these violations, a Heaviside function was introduced to separate the follow‐up time into before and after 10 years post‐T2DM diagnosis for nephropathy, and before and after 15 years for smoking and stroke.

After determination of the association of each potential risk factor and the development of a foot complication in a univariate model, a time‐dependent multivariate Cox model was established using a backward elimination approach. The critical alpha values for the elimination of variables were chosen in accordance with the Akaike information criterion (AIC).^30^ To evaluate the robustness of the Cox model, an alternative Cox model was built using a forward selection strategy by consequentially adding all risk factors with *p*‐values <.1. This threshold was used as a trade‐off in order to prevent the missing of important risk factors while at the same time limiting irrelevant risk factors.

## 3 RESULTS

### 3.1 Patient characteristics

**FIGURE 1:**
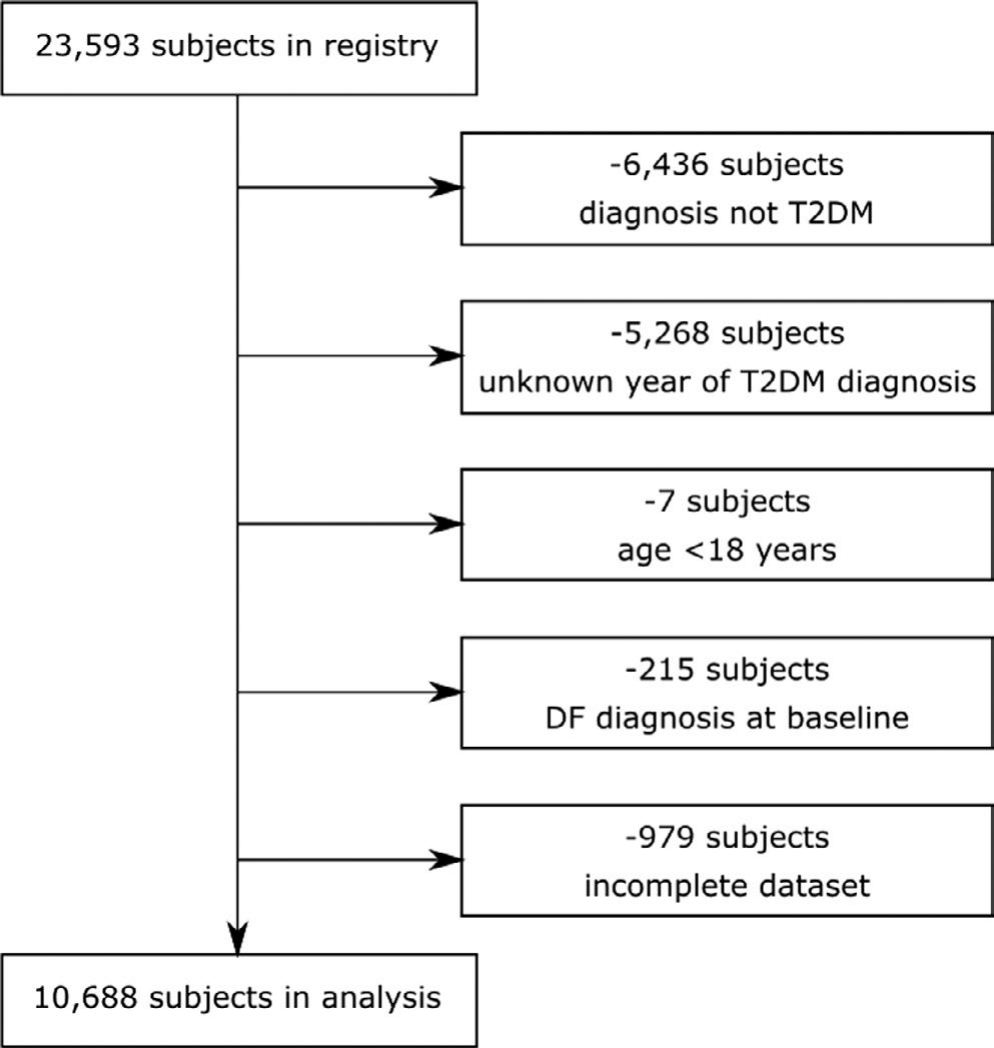
Consort diagram showing patient selection. Abbreviations: DF, diabetic foot; T2DM, type 2 diabetes mellitus

In total, 10,688 out of 23,593 patients fulfilled the predefined inclusion criteria and were included in the analysis. The flow diagram of the patient selection is depicted in Figure 1.

The overall mean (±SD) age at diagnosis was 63.21 ± 12.58 years, and 44.3% were female. Overall, 140 DF events occurred during a mean follow‐up period of 9.75 years.

Baseline characteristics of the study population are depicted in Table 1. Patients who sustained a DF complication were less frequently female than male (30.0% vs. 44.5%) and had a higher level of HbA1c (8.3% [SD 1.7] vs. 7.7% [1.5]) compared to patients who did not sustain a DF event. In addition, patients with DF conducted foot inspections more often. The use of insulin or insulin analogues was documented more often for patients with DF compared to patients without any DF event. Concerning other late complications related to diabetes, prior diagnoses of nephropathy, retinopathy, neuropathy, myocardial infarction, stroke and peripheral arterial disease or coronary bypass/PTCA were more frequently present in the group of patients with DF. However, no statistically significant differences were evident between the two groups concerning age at diagnosis, smoking status, physical activity, BMI, hypertension and participation in education programmes.

**TABLE 1:** Demographic and clinical characteristics for cohort without DF vs. cohort with DF

Variable	no DF (*N* = 10,548)		DF (*N* = 140)		*p*‐Value
	*N* or mean ± SD	%	*N* or mean ± SD	%	
Age at diagnosis [years]	63.2 ± 12.6		63.0 ± 11.9		.888
≤50 years	1,691	16.0	23	16.4	.977
>50 years and ≤70 years	5,609	53.2	75	53.6	
>70 years	3,248	30.8	42	30.0	30.0
Female gender	4,694	44.5	42	30.0	.001
Active smokers	1,976	18.7	26	18.6	.961
Nephropathy	1,568	14.9	57	40.7	<.001
Retinopathy	225	2.1	9	6.4	.001
Neuropathy	811	7.7	58	41.4	<.001
Myocardial infarction	975	9.2	31	22.1	<.001
Stroke	654	6.2	16	11.4	.011
Peripheral arterial disease	403	3.8	34	24.3	<.001
Coronary bypass/PTCA	897	8.5	32	22.9	<.001
Physically active	5,643	53.5	71	50.7	.512
Increased BMI (≥30 kg/m²)	4,539	43.0	63	45.0	.640
Hypertension (≥140/90 mmHg)	3,977	37.7	54	38.6	.833
HbA1c [%]	7.7 ± 1.5		8.3 ± 1.7		<.001
<6.5%	2,343	22.2	15	10.7	<.001
≥6.5% and ≤9%	6,585	62.4	89	63.6	
>9%	1,620	15.4	36	25.7	
Insulin or insulin analogues use	5,415	51.3	102	72.9	<.001
Participation in education program	7,945	75.3	111	79.3	.280
Conduction of foot inspection	4,443	42.1	73	52.1	.017

Note

Significance defined as *p* < .05. Continuous variables expressed as mean (± standard deviation).

Abbreviations: BMI, body mass index; DF, diabetic foot; PTCA, percutaneous transluminal coronary angioplasty; SD, standard deviation.

### 3.2 Risk factors for DF

In the univariate Cox model, the most strongly associated variable with DF was peripheral arterial disease with a hazard ratio of 4.50 [95%CI: 3.03–6.68]. Other statistically significant associations with DF were found in male gender, myocardial infarction, neuropathy and coronary bypass/PTCA. Increased age at diagnosis was associated with a reduced risk of DF events (hazard ratio 0.51 [0.30–0.88] for >70 years vs. ≤50 years). In addition, no difference was seen for nephropathy in the first 10 years after initial diagnosis of T2DM, while a statistically significant association was found after 10 years (hazard ratio 2.77 [1.83–4.20]). The levels of HbA1c were analysed separately for the first 15 years after initial diabetes diagnosis and for the time after 15 years. When comparing HbA1c values of >9% and <6.5% in the time later than 15 years after diabetes diagnosis, a hazard ratio of 4.09 [0.95–17.63] was retrieved (*p*‐value = .059). Increased BMI yielded a hazard ratio of 1.38 [0.99–1.94], reaching borderline significance (*p*‐value = .061). The results of the univariate analysis are shown in Table 2.

**TABLE 2:** Hazard ratios of diabetic foot complications (time‐dependent univariate analysis)

Variable	Hazard Ratio	95% CI	*p*‐value
Age at diagnosis			
≤50 years	Reference		
>50 years and ≤70 years	0.65	0.40–1.06	.082
>70 years	0.51	0.30–0.88	.014
Male gender	2.01	1.39–2.92	<.001
Active Smokers	1.16	0.75–1.79	.501
Nephropathy			
t ≤10 years	1.15	0.57–2.33	.693
t >10 years	2.77	1.83–4.20	<.001
Retinopathy	0.88	0.41–1.88	.735
Neuropathy	3.82	2.70–5.42	<.001
Myocardial infarction	1.80	1.19–2.72	.005
Stroke			
t ≤15 years	1.02	0.44–2.34	.967
t >15 years	1.84	0.90–3.76	.094
Peripheral arterial disease	4.50	3.03–6.68	<.001
Coronary bypass / PTCA	1.89	1.26–2.84	.002
Physical activity	0.87	0.62–1.22	.407
Increased BMI (≥30 kg/m²)	1.38	0.99–1.94	.061
Hypertension (≥140/90 mmHg)	0.99	0.70–1.40	.965
HbA1c (t ≤15 years)			
<6.5%	Reference		
≥6.5% and ≤9%	0.62	0.33–1.15	.126
>9%	1.05	0.51–2.15	.898
HbA1c (t >15 years)			
<6.5%	Reference		
≥6.5% and ≤9%	1.80	0.43–7.46	.421
>9%	4.09	0.95–17.63	.059
Insulin or insulin analogues use	0.74	0.51–1.09	.128
Participation in education program	0.89	0.59–1.34	.583
Conduction of foot inspection	1.06	0.76–1.49	.735

Note

Significance defined as *p* < .05.

Abbreviations: BMI, body mass index; CI, confidence interval; PTCA, percutaneous transluminal coronary angioplasty.

**FIGURE 2:**
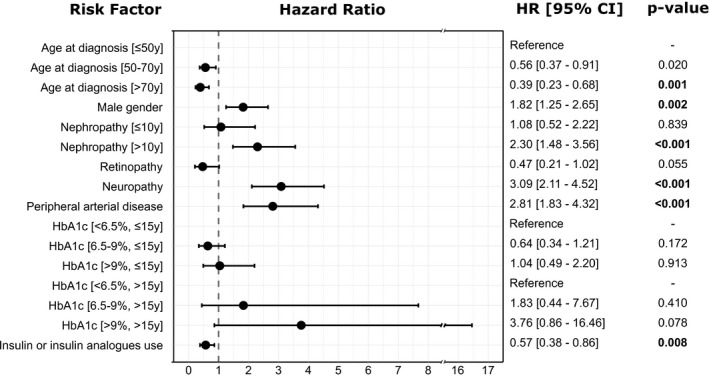
Predictors of diabetic foot complications (time‐dependent multivariate analysis). Significance defined as *p* < .05. Abbreviations: CI, confidence interval; HR, hazard ratio; y, years

A time‐dependent multivariate Cox model was used to analyse independent risk factors for the initial development of DF complications. The final results showed that neuropathy (hazard ratio 3.09 [2.11–4.52]), peripheral arterial disease (2.81 [1.83–4.32]), nephropathy after 10 years post‐T2DM diagnosis (2.30 [1.48–3.56]) and male gender (1.82 [1.25–2.65]) were associated with an increased risk for DF events. Increased age at diagnosis (0.56 [0.37–0.91] for >50 years and ≤70 years vs. ≤50 years, and 0.39 [0.23–0.68] for >70 years vs. ≤50 years) and use of insulin or insulin analogues (0.57 [0.38–0.86]) was associated with a lower likelihood of DF events. Again, a high hazard ratio (3.76 [0.86–16.46]) was retrieved for the association between HbA1c levels >9% and <6.5% in the time after 15 years after the diabetes diagnosis. However, the level of statistical significance was not reached (Figure 2).

To evaluate the robustness of the multivariate Cox model developed by means of a backward elimination approach, an alternative model was built using a forward approach. In this second multivariate model, the same set of independent risk factors for DF development was identified, highlighting the robustness of the analysis.

## 4 DISCUSSION

Given the tremendous personal and financial burden associated with foot complications in patients with T2DM, the need for better understanding of this late complication is critical for its prevention. We have performed a large registry‐based retrospective cohort study in a population of T2DM patients without previous foot complications. In this study, a prevalence of DF of 1.31% was reported, a value that lies below the ranges previously reported for European countries (1.7–4.8%).^31^ However, this may derive from the fact that all patients with foot complications present at the first visit recorded in the registry were excluded from the analysis in order to take only information prior to DF diagnosis into account.

In the time‐dependent multivariate Cox regression model, neuropathy, peripheral arterial disease, nephropathy after 10 years after T2DM diagnosis, and male gender were identified as significant and independent risk factors for DF development. In contrast, higher age at diagnosis and use of insulin or insulin analogues were identified as having a protective effect. Discrepancies between the set of risk factors identified in the univariate models and in the multivariate model highlight the presence of confounding variables. The results of an alternative multivariate model built with a forward selection approach provided reliability concerning the robustness of the model built with backward elimination.

Due to their described role in the development of foot complications in general, a strong association between peripheral neuropathy and peripheral arterial disease and the development of DF is consistent with the literature.^20^, ^32^ Peripheral neuropathy was identified as the strongest risk factor for foot complications in this studied patient cohort. These findings are consistent with other studies that were using age and gender adjusted multivariate logistic regression models and in which the association has also been reported.^19^, ^33^, ^34^ This strong association is likely to reflect the high level of nerve damage present in the lower extremities of diabetic patients, which highlights the need for increased screening for lower extremity nerve defects in this patient population. Peripheral arterial disease was the second strongest factor associated with DF in this study. Given the fact that ischemia causes tissue damage and leads to poor wound healing, peripheral arterial disease is known as an important risk factor in the pathogenesis of foot complications.^8^, ^35^ The crucial role of peripheral arterial disease as a risk factor for DF development has been highlighted by various previous studies.^33^, ^34^, ^36^

Other diabetes‐related complications, namely nephropathy and previous myocardial infarction or coronary bypass/PTCA were significantly associated with foot complications in the univariate model. However, when adjusting for other potential risk factors in the time‐dependent multivariate model, the association remained significant only with nephropathy. While for the first 10 years after the diabetes diagnosis, no statistically significant association was seen in patients with nephropathy, the risk for developing a DF event is 2.30 times higher in such patients later than 10 years after the diabetes diagnosis. Nephropathy might be anticipated as a risk factor for DF due to the common physiological origin of microvascular late complications.^4^ However, in previous studies, the role of nephropathy as a risk factor remained inconsistent: While several groups identified a positive association,^34^, ^36^, ^37^ no association was retrieved in other studies.^19^, ^38^, ^39^ To the knowledge of the authors, however, there is no study available in which the association between nephropathy and DF was analysed in separate time intervals.

Male gender was identified as a strong predictor for DF in the multivariate Cox model. The identified risk of DF development is almost doubled in male patients compared to female patients. A similar effect was shown consistently in various other studies.^19^, ^33^, ^37^ This effect may be explained to some extent by the higher foot pressure found in male patients, probably due to higher mean height in men compared to women.^40^ In addition, women are known to be more active in terms of self‐care and preventive care concerning diabetic foot lesions, whereas men show a more passive attitude.^41^

Higher age has been identified as having a protective effect on the DF development (i.e. with increasing age at diagnosis, the hazard of foot ulcer was found to decrease). This effect was not only seen when comparing >70 years and ≤50 years of age at diagnosis, but also when age between 51 and 70 years was compared to ≤50 years. Additionally, other groups reported a negative association between higher age and different endpoints related to foot complications: Abbott et al. and Dekker et al. reported a negative association between age and foot ulcer development (hazard ratio 0.957 and odds ratio 0.991 for every year increase, respectively),^34^, ^42^ and Yang et al. identified the same relationship when analysing lower extremity amputation as the endpoint of interest (odds ratio 0.8 for age ≥65 years compared to younger patients).^43^ However, as highlighted in two recent systematic reviews on risk factors for DF development, the results on the potential association between age and DF are highly contradictory.^17^, ^18^ While in several studies, a positive association was identified between higher age and various endpoints such as foot ulcers and lower extremity amputations,^19^, ^38^, ^39^ other groups did not find any association.^33^, ^35^, ^36^, ^37^ There are different hypotheses that aim to explain the protective effect of age at diagnosis: A possible explanation is that older patients with severe courses of disease, that render them immobile, might be underrepresented in the registry. This selection bias would lead to the possibility that the older patient groups represented in the registry are those who are healthier.^42^ Dekker et al. furthermore hypothesized that younger patients are more physically active compared to older patients and are therefore more prone to traumatic situations which increases the risk of foot ulcers.^34^ However, further studies are needed to gain more detailed insights on the relationship between age and DF development.

A protective effect was detected concerning the use of insulin or its analogues. This finding is not in line with several studies that identified a positive association between insulin use and subsequent foot complications^19^, ^37^, ^44^ or did not find any association.^45^ However, in the cohort study on hand, potential risk factors have only been analysed prior to the development of a foot complication, whereas in several other studies, patients with DF were compared to patients without DF. Therefore, a positive association could derive from the fact that patients who are already receiving treatment for foot complications are more likely to be insulin users.^19^, ^44^ In addition, insulin treatment prior to DF development might improve glycaemic control, thereby preventing subsequent late complications. Nonetheless, further prospective studies are required to investigate this association in more detail.

Although the level of statistical significance was not reached in the multivariate analysis, a hazard ratio as high as 3.76 for HbA1c values >9% compared to <6.5% in the time after 15 years after the diabetes diagnosis might be of high clinical relevance. A positive association between HbA1c levels and foot complications was shown by several groups, ^19^, ^36^, ^39^ while others could not find any association.^37^, ^45^ Our data suggests that since glycaemic control can be altered by lifestyle changes and/or treatment modalities, improving glycaemic control might be beneficial to reduce the risk of subsequent DF events. Another modifiable risk factor, that is, the patient's BMI, showed a hazard ratio of 1.38 in the univariate model, and although this factor was not statistically significant, it might still be clinically relevant. Various studies on the association between BMI and subsequent foot complications have come to discordant results ranging from positive associations to no effects at all to a protective effect of higher BMI.^18^ When considering the same level of mobility, there is higher pressure on the lower extremities in obese patients compared to patients with a lower BMI. This is proposed to be linked to more frequent DF events.^40^ However, there is still a lack of consensus regarding the relationship between BMI and foot complications.^46^ Additional studies are thus required to clarify to this relationship. From a public health perspective, a reduction in overweight is anticipated to be beneficial for the patients’ overall health independent of potential foot complications.

Our study is characterized by several strengths and weaknesses. The main strength of this multicentre study lies in its large sample size with a wide range of demographic, clinical and behavioural data. The DRT is a region‐wide registry covering all ten hospitals and eight outpatient practices in Tyrol. Further strengths include data collection by specialized personnel and the fact that the cohort is derived from a large web‐based electronic registry focussing on diabetes and its complications with regular follow‐ups and data validation. The study population is well defined by the clinical confirmation of the T2DM diagnosis. Furthermore, since all patients with foot complications at baseline were excluded from the analysis, and only data prior to the year of DF development were analysed, it was ensured, that the association of potential risk factors present prior to a potential DF event were assessed in the analysis. Despite the limited number of DF events in the patient cohort, our study design allows for the establishment of cause–effect relationships between pre‐existing patient characteristics and subsequent foot complications. The thorough evaluation of the proportional hazard assumption and the development of a time‐dependent multivariate Cox regression model including the test for robustness using an alternative modelling approach allowed for the analysis of risk factors independent of potential confounders and the validation of the robustness of the model.

However, due to the hospital‐ and outpatient practice‐based setting, a certain degree of selection bias might persist. The nature of such biases cannot be determined, and hence, cannot be adjusted for. Both, mild and very severe courses might be underrepresented within the registry, however, to a varying extent. While diabetic patients with mild courses of disease might be treated solely by their general practitioners, patients with very severe courses might not be included due to highly reduced mobility. Of note, this study was solely observational and is based on real‐world data. Without a strictly defined study environment and with data being collected within the routine setting, clinical processes and procedures could not be altered, and consequently, diagnostic methods and therapeutic decisions could not be harmonized. Therefore, while the external validity for diabetic patients within a hospital setting is suggested to be high, it remains unknown for the general population of patients with T2DM. Moreover, the care for diabetic patients and their paths through the health care system are not clearly structured in Austria and are not organized by the state health system, which can result in differences concerning the time points at which patients are referred from general practitioners to hospitals and therefore, differences in their modes of treatment. In addition to the selection bias, recall bias could blur information that is collected retrospectively from the patients such as the year of diabetes diagnosis. The exclusive analysis of foot ulcers defining the endpoint within this study might yield a certain underestimation of foot complications. Furthermore, since the registry data is collected during the routine setting, a form with predefined data fields was found to be the best solution to unify the data to the greatest possible extent. However, it cannot be ruled out that some bias is introduced by physicians’ varying interpretation of the requirements for each data field. Furthermore, since certain diagnoses such as retinopathy and in part neuropathy are established by an external specialist with no access to the registry data form, an underrepresentation of such diagnoses might have occurred. Additionally, it has to be emphasized that due to the predefined set of variables, the volume of data that is collected within the registry is limited, which leads to a lack of clinical detail and the potential of residual confounding.

## 5 CONCLUSION

We have conducted a large retrospective cohort study to investigate the association between various potential risk factors and subsequent initial development of DF complications in patients with T2DM. Our study revealed statistically significant associations of DF with neuropathy, peripheral arterial disease, nephropathy and use of insulin or insulin analogues. Moreover, demographic characteristics such as age at diagnosis and gender were shown to play an important role in the risk for DF. We therefore suggest that readily available information on the patients’ demographic data, medical history and comorbidities may facilitate personalized screening. Large longitudinal studies are needed to investigate whether the reduction in existing risk factors leads to a decrease in the number of subsequent foot complications in patients with T2DM.

## CONFLICTS OF INTEREST

All authors declare that there is no conflict of interest.

## AUTHOR CONTRIBUTIONS

SR developed the study protocol, performed the statistical analysis, and wrote the manuscript. BR was involved in the conduction of the statistical analysis and the design of the tables and figures. HS and ML contributed their clinical expertise to writing the manuscript. WO was involved in the study design, the conduction of the analysis and writing the article. All authors have read and approved the final manuscript.

## ETHICAL APPROVAL

The present study was approved by the Ethics Committee of the Medical University of Innsbruck as part of the Diabetes Registry Tyrol. Due to the retrospective nature of the study, no additional ethics committee approval is required by the Austrian law. This was confirmed by the Ethics Committee of the Medical University of Innsbruck via a general statement. Procedures concerning patients and data collection, handling and storage of personal data are conducted in accordance with national laws and the ethical standards of the seventh revision of the Declaration of Helsinki. Furthermore, the study was approved by the Research Committee for Scientific Ethical Questions at the UMIT, the Private University for Health Sciences, Medical Informatics and Technology.

## DATA AVAILABILITY STATEMENT

The data that support the findings of this study are available from the Diabetes Registry Tyrol. Restrictions apply to the availability of these data, which were used under license for this study. Data are available from the Diabetes Registry Tyrol with the permission of the Department of Clinical Epidemiology (Tirol Kliniken, Innsbruck, Austria).
